# A Woman’s Place Is in Theatre, but Are Theatres Designed with Women in Mind? A Systematic Review of Ergonomics for Women in Surgery

**DOI:** 10.3390/jcm11123496

**Published:** 2022-06-18

**Authors:** Maria Irene Bellini, Maria Ida Amabile, Paolina Saullo, Noemi Zorzetti, Mario Testini, Roberto Caronna, Vito D’Andrea

**Affiliations:** 1 Department of Surgical Sciences, Sapienza University of Rome, 00161 Rome, Italy; mariaida.amabile@uniroma1.it (M.I.A.); paolina.saullo@gmail.com (P.S.); noemi.zorzetti@gmail.com (N.Z.); mario.testini@uniroma1.it (M.T.); roberto.caronna@uniroma1.it (R.C.); vito.dandrea@uniroma1.it (V.D.); 2 Academic Unit of General Surgery, Department of Biomedical Sciences and Human Oncology, School of Medical, University of Bari Aldo Moro, 70120 Bari, Italy

**Keywords:** diversity and inclusion, gender equity, ergonomics, surgical education and training, female surgeons

## Abstract

Background: Literature regarding ergonomic protocols for surgery is lacking, and there is a paucity of information on how this impacts on gender differences with regards to the barriers faced by women in surgery. Methods: This article reviews current literature addressing women in surgery and ergonomics through a systematic search including the Web of Science, Scopus, and PubMed databases. Results: Searches retrieved 425 items, and after a thorough evaluation for inclusion, 15 studies were examined—predominantly surveys (n = 9) and originating from the USA (n = 9). Identified ergonomic challenges included the general shorter height and smaller glove size of women. Furthermore, women experienced more musculoskeletal pain than men, potentially because the size and design of theatre tools are designed for male and tall individuals, highlighting an unconscious gender bias still pervading the surgical field. Conclusions: As more women enter medicine and pursue surgical careers, it is essential to foster a culture of diversity and inclusion in theatre to develop more ergonomic environments.

## 1. Introduction

Surgery remains overwhelmingly a male field [1]. Despite the fact that efforts to attract and retain the female surgical workforce are growing daily, these focus mainly on promoting a change in the culture [2], which has fortunately already resulted in a significant reduction in much of the overt sexism that was apparent in previous years. However, there remains an overarching theme where unconscious bias identifies surgeons to a stereotype of male individuals [3].

Initiatives aiming at how to strategically tackle gender equity among professionals, through networking and mentoring supported by societies and organisations, are in place [4,5,6,7], but not much has been done to relieve the struggle commonly associated with long operations. There is evidence that female surgeons experience more discomfort in their bodies and their hands than their male colleagues [8,9,10].

It comes then as no surprise that the abovementioned issues add to the well-known surgical work–life imbalance, with a direct impact on women’s ability to work, to operate, and to acquire practical skills. Altogether, these factors are likely to affect the determination of prospective female trainees, and to negatively impact on the retention of the current female surgeons.

Why are diversity and inclusion (DEI)—i.e., the recognition that each individual requires different resources and opportunities to achieve an equal outcome—so important? In general, it is thought that people with different experiences, perspectives, and thinking styles combine and collaborate to create a stronger, more successful environment, so the inclusion of diverse surgical members ultimately builds better teams [3,4].

The aim of this review is to identify the principal barriers perceived by women according to the “ergonomics” principles, i.e., the scientific discipline concerned with the understanding of interactions between humans and other elements of a system, and the field that applies theory, principles, data, and methods to design, aiming to optimise human wellbeing and overall system performance [11].

## 2. Materials and Methods

**Figure 1:**
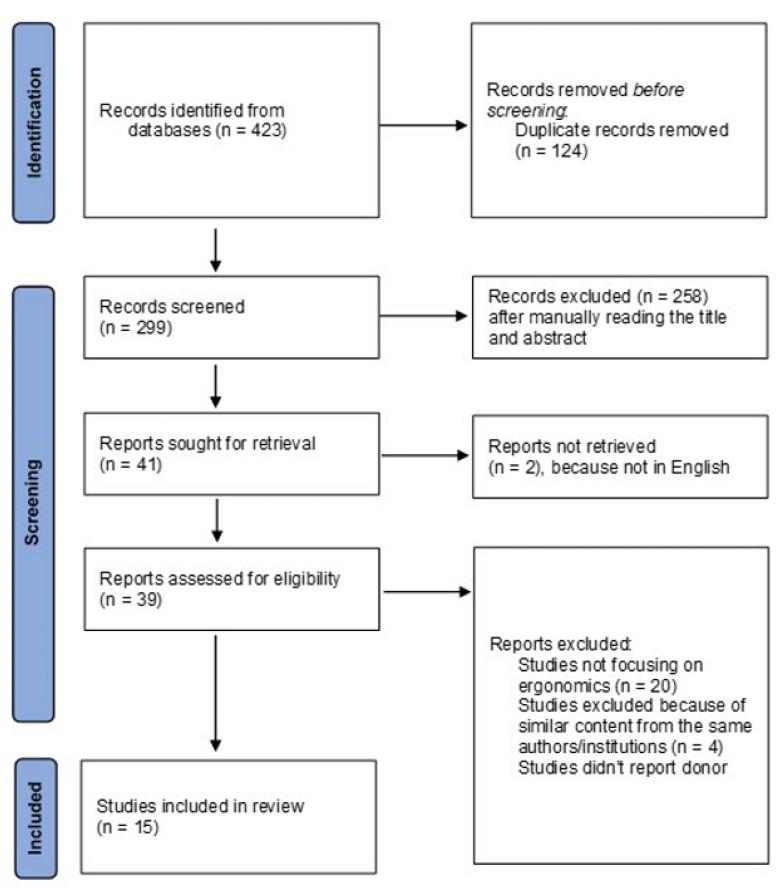
PRISMA flow diagram.

A systematic search was performed to identify studies focusing on ergonomics for women in surgery within the PubMed, Scopus, and Web of Science electronic databases. Only original articles in English, with no time restrictions, and reporting specifically on the principles of ergonomics—as previously identified [11]—were considered. Search terms included combinations of the keywords “ergonomics” and “women in surgery”. Studies that originated from the same centre were considered only if not overlapping in the reported cases. Two independent reviewers (M.I.B. and M.I.A.) performed the search and the screening, before producing a list of studies eligible for inclusion. In case of disagreement, a third reviewer (P.S.) was consulted. The included studies were evaluated according to the Newcastle–Ottawa Scale [12]. The complete evaluation is reported in Table 1. The PRISMA diagram for the search is reported in Figure 1.

## 3. Results

The systematic search identified the following results in each of the investigated databases: Web of Science (n = 73), PubMed (n = 273), and Scopus (n = 77). After duplicate removal (n = 124) and screening based on titles and abstracts, a total of 41 articles were analysed. Reading those articles in full then led to 15 studies meeting the inclusion criteria. Most studies (n = 9) were surveys, and with the USA as the country of origin (n = 9). The specialties represented were general surgery (n = 7), gynaecology (n = 4), dentistry (n = 1), endocrine surgery (n = 1), orthopaedics (n = 1), and otorhinolaryngology (n = 1). A full list of the studies included for review is summarised in Table 2; the main findings are described below.

### 3.1. Work-Related Physical Discomfort

The incidence of physical strain secondary to maintaining prolonged uncomfortable postures, or to remaining static holding retractors with high levels of manual force, has been reported frequently [15,19,22,23]. More specifically, we can distinguish between musculoskeletal symptoms and hand symptoms. The regions of interest are the cervical (58.1%), dorsal (40.5%), lumbar (52.7%), wrist (27.1%), and shoulder regions (24.3%) [19], although the frequency might vary according to the type of surgery performed.

Female sex per se is linked to a more severe progression of upper limb musculoskeletal disorders; in fact, the prevalence of rotator cuff syndrome is reported to be as high as 6.6% in men and 8.5% in women, on a general population basis [25]. In the literature, women reported experiencing pain or discomfort associated with their surgical practice [13,16,21], with an approximately twofold risk in comparison to their male colleagues [13]. Shortness is also associated with higher discomfort [14,24], particularly when performing minimally invasive surgery; shorter surgeons are in fact at greater risk of spinal torsion to watch the monitor, given their propensity to pose their shoulder flexed, thus leading to greater spinal rotation to the right. Interestingly, in the study by Stewart et al. [24], greater pain scores were related to short stature (*p* < 0.001), i.e., height ≤ 168 cm, and male gender (*p* < 0.001). The use of robotic system seems to reduce pain scores in comparison to both laparoscopy and open surgery [17,24], or to cancel any significant pain-related effects, although this is limited only to certain demographic subgroups [24], i.e., tall surgeons (*p* = 0.07) and female surgeons (*p* = 0.13).

### 3.2. Compliance of Surgical Devices with Female Requirements

In laparoscopy, the evaluation of wrist movements is also of utmost importance, given the need for long levers and pistol-grip handles, causing poor force transmission efficiency from the forearm to the hand. Three studies [9,10,21] examining surgeons with small glove sizes (i.e., <6.5) reported a low satisfaction level regarding the anastomotic staplers, as well as when using laparoscopic instruments in general. These findings suggest an unaccommodating environment, in which the usability of surgical tools is significantly compromised by different hand sizes and finger lengths. Furthermore, firing the stapler by gripping the proximal side of the lever was defined as “physically impossible” for most women with small glove sizes [9]. Kono et al. reported that the most appropriate stapler diameter for surgeons with a given hand size is not the same for male and female individuals, but needs to be established separately for each sex, ideally by developing smart instruments whose usability is not affected by the gender identity of the user [22]. This also applies to the ergonomics of handles for laparoscopic tools in general [16].

### 3.3. Impact on Training, Productivity, and Career Longevity

A positive correlation has been reported between higher pain scores and lower work satisfaction, burnout, and callousness toward others [23], meaning that poor ergonomics are not only an obstacle to progress in training and advancement in the surgical career, but also have a negative impact on leisure activities [13] and productivity [23], thus further decreasing the attractiveness of surgical specialties. However, self-selected settings were demonstrated to be improved by following objective surgeon postures, for both women and men [25], with low percentages of respondents reporting having received ergonomics education during surgical training [21].

## 4. Discussion

Among surgical specialties, a tacit acceptance of physical efforts in addition to the mental concentration required to perform complex operations has encouraged stoicism—particularly in the form of not reporting work-related injuries [13]. However, it is part of human nature to feel fatigue when operating for long periods without breaks, and it should not be labelled as a sign of weakness to seek alternatives, or to disclose the struggles that surgeons have been through. On the contrary, this review highlights the need to improve ergonomic challenges in operative settings so as to not discourage those who naturally have a different stature or muscular capacity in terms of their advancement or their feeling of belonging to the surgical field. This would really imply a true DEI environment in the operating theatre.

As already mentioned, women in general tend to have significantly less absolute and relative skeletal muscle mass [26], but nevertheless, they should not feel “unwelcome” to pursue a surgical career. Indeed, on the contrary, it is a sign of strength to recognise one’s own limitations; furthermore, for organisations to achieve true gender equity, important consideration must be given by providing women with the same opportunities to succeed, following the principles of DEI.

What could be the initial changes to adopt? Ergonomics education during training should be prioritised [27], as often younger colleagues might be at higher risk of muscular strain [28]. To assume the assistant role, in fact, often entails adapting to contorted body positions for holding instruments with prolonged retraction or, for shorter surgeons, reaching the table height might require them to use a step stool, which contributes to awkward postures to keep the surgical field in view.

It would be recommendable, therefore, to adjust the height of the monitor to a common level of suitability for the whole operating staff; eventually, the use of a second screen appears essential in considerations of different surgeons’ heights, aiming to place the screen at the centre, just below the eye level.

With regards to the instruments, as noted in the present review [9,10,18,22], there is evidence that an adjustment according to hand size—especially for those with smaller glove sizes, mainly female—is a priority so as to not discriminate in surgical practice. Unsurprisingly, two studies were from Japan, where the 2018 exposé of Tokyo Medical University [29]—which had reduced the scores of its female applicants over a period of at least 12 years to cap female entrants at 30%—revealed the regressive thinking unfortunately hindering medical practice in that country. Luckily, a more legal framework is now in place to prevent discrimination. Importantly, a more diverse staff also helps patients from minority backgrounds to feel better valued during their hospital stay. In fact, it is not uncommon to observe—because of religious and sociocultural beliefs—challenges for women to be assisted by male doctors or associated staff; therefore, to have an impact on patient health outcomes and quality of life, the care the same patients experience and how they perceive their representation is essential [30,31].

Additionally, a relatively low number of females generally participated in most of the analysed studies, highlighting the need for gender-focused research—especially in surgery, where the field is still male-dominated. Interestingly, according to a large Canadian registry analysis, patients treated by female surgeons had a decrease in 30-day mortality, length of stay, complications, and readmission, in comparison to those treated by male surgeons. These important findings could be the result of a higher level achieved by women who successfully completed their training, in view of a more uncomfortable environment that may have significantly impacted on the development of their performance and skills [32].

To overcome physical-related challenges, several solutions have been proposed, such as the use of chairs providing chest support and adjustable armrests, as well as footrests mounted with a bipolar control pedal. Those chairs, motor-driven and adjustable according to the patient’s and surgeon’s size, could be personalised, with the result of ergonomic improvement during excision and suturing, allowing the operation to be safely transferred to second generation of surgeons for training purposes [33]. The combination of standing with sitting would be also beneficial to lower down the risk of developing chronic venous disorders, known to be higher in professionals working in prolonged sitting or standing postures [34]. Operating surgeons should also be encouraged to take minibreaks during long operations, or to eventually perform stretching as a relief from the musculoskeletal grief accumulated. It is therefore highly recommendable to raise awareness of ergonomics for all theatre staff, aiming to optimise human wellbeing and overall system performance [11]. From the present review, educational interventions such as the one reported by Hokenstad et al. [20] improved objective surgeon posture at the console, when compared to the previous surgeons’ self-selected settings, demonstrating room for improvement towards higher surgeon awareness.

Another valuable solution could be the use of automated and personalised theatre tools, such as regulable laparoscopic instruments or smart technology able to read and translate tissue characteristics, thus alerting surgeons when and where to clip or to staple. The artificial intelligence applied in this way would allow, for instance, that staplers or clip applicators could be fired and handled by anyone, independently from the force a particular individual could provide.

The relatively low number of studies focusing on ergonomics for women in surgery limited the level of evidence we were able to achieve, based mainly on survey data and small sample size studies.

In conclusion, future research should focus on the mitigation of surgeons’ physical-related barriers and seek to understand the ergonomic gender differences that could act as an impediment during training and progression in the surgical career. This is of utmost importance in a context of full inclusivity, so as to acknowledge diverse medical device design and integrate technology and innovation to optimise human wellbeing and the overall system performance.

## Figures and Tables

**Table 1:** Quality of evidence in the selected papers for the systematic review according to the Newcastle–Ottawa Scale.

Author	Selection	Comparability	Outcome	Total
Adams et al. [13]	2			2
Aitchison et al. [14]	3		1	4
Armijo et al. [15]	3		1	4
Berguer et al. [10]	2			2
Cavanagh et al. [16]	2			2
Dalsgaard et al. [17]	3		1	4
Gonzalez et al. [18]	3		1	4
Harutunian et al. [19]	2			2
Hokenstad et al. [20]	3		1	4
Jensen et al. [21]	2			2
Kono et al. (2012) [22]	2			2
Kono et al. (2014) [9]	3		1	4
McQuivey et al. [23]	2			2
Stewart et al. [24]	2			2
Sutton et al. [8]	2			2

**Table 2:** Study characteristics, aims, and main findings.

Study	Specialty	Country	Method	Percentage of Female Participants (Female/Total Participants)	Findings
Adams et al. [13]	Gynaecology	USA	19-item survey on demographics, surgical practice patterns, physical symptom duration, and effect on work or leisure activities	49.7% (246/495)	Musculoskeletal symptoms are highly prevalent, and female sex is associated with an approximately twofold risk of reported pain in the lower back region (*p* = 0.02), in the upper back (*p* = 0.002), and in the wrist/hand region (*p* = 0.001)
Aitchison et al. [14]	Gynaecology	Australia	Observational: video recordings of 18 surgeons	83.3% (15/18)	Shorter surgeons maintain significantly greater degrees of neck rotation when looking at the monitor (*p* < 0.003). Surgeons with shorter arm lengths spend longer time in extreme positions with their non-dominant shoulder at >90° (*p* = 0.04) and elbow at >120° (*p* < 0.001)
Armijo et al. [15]	General surgery	USA	Observational: evaluation of muscle group activation during surgery via electromyography	44.4% (8/18)	Increase in muscle activation is observed for female laparoscopic surgeons (*p* < 0.001). Self-perceived sensory (*p* = 0.026) and cognitive (*p* = 0.045) fatigue scores are higher among female surgeons at the end of the surgery.
Berguer et al. [10]	General surgery	USA	Online survey on demographic and practice data, musculoskeletal symptoms, and the perceived difficulty in using several types of laparoscopic instruments	21.9% (159/726)	Hand size is a significant determinant of difficulty using laparoscopic surgical instruments, particularly for sizes 6.5 or smaller (*p* < 0.001)
Cavanagh et al. [16]	Otorhinolaryngology	USA	28-item online survey on demographics, surgical practice characteristics, physical symptoms, and ergonomics	15.0% (15/100)	Female surgeons report higher experience of pain/discomfort associated with their surgical practice (*p* = 0.033)
Dalsgaard et al. [17]	Gynaecology	Denmark	Observational (semi-randomised): bipolar surface electromyogram; calculation of gaps per minute plus static and peak muscle activation were calculated during surgeries	58.3% (7/12)	Neck and static shoulder muscle activities are lower in robotic surgery compared to laparoscopy (*p* < 0.05)
Gonzalez et al. [18]	General surgery	Spain	Observational: Trial to determine the optimal diameter of the handle from an ergonomic point of view	51.1% (69/135)	The optimal diameter of the instrument’s handle differs according to the hand size, especially for smaller hands (*p* < 0.05)
Harutunian et al. [19]	Dentistry	Spain	19-item survey on demographics and questions regarding ergonomics of the instrument holder and resulting musculoskeletal disorders	52.7% (39/74)	Most of the dentists experience musculoskeletal pain, and women show a higher frequency of intense pain (*p* < 0.05)
Hokenstad et al. [20]	Gynaecology	USA	Inertial measurement and survey before and after ergonomic implementation during robotic hysterectomy	50.0% (3/6)	Improved objective surgeon posture at the console when compared with the surgeons’ self-selected settings: neck (*p* = 0 .008) and right upper arm (*p* = 0.02)
Jensen et al. [21]	Endocrine surgery	USA	43-item online survey on demographics, surgical information, prevalence of musculoskeletal symptoms, and pursued therapy/treatment; ergonomic recommendations	32.9% (72/220)	Women more likely to report pain and stiffness after surgery (*p* = 0.004). Most common locations are the neck and shoulder
Kono et al. (2012) [22]	General surgery	Japan	9-item online survey on demographics and questions regarding circular and linear staplers	30.4% (74/243)	Surgeons with small glove sizes express a low satisfaction level regarding the anastomotic staplers (*p* < 0.0001), suggesting a need to develop instruments whose usability is not affected by different hand sizes and lengths of fingers
Kono et al. (2014) [9]	General surgery	Japan	Observational: evaluation of the relationship between grip width and the operation force required to push the lever of the stapler	53.7% (61/113)	Men have wider optimal grip width than women for both the dominant and non-dominant hands (*p* < 0.001)
McQuivey et al. [23]	Orthopaedics	USA	Online survey on demographics, symptoms by body part, and attitudes/beliefs/behaviours regarding surgical ergonomics	27.6% (21/76)	No sex-specific differences, but concerns about implications for work satisfaction (*p* = 0.005), burnout (b = 0.04), and callousness toward others (*p* < 0.0001)
Stewart et al. [24]	General surgery	USA	Survey on demographics, the surgery performed, intraoperative ergonomics, and task load during surgery	28.2% (24/85)	Short surgeons and male surgeons report more pain after both open and robotic operations (*p* < 0.001).
Sutton et al. [8]	General surgery	USA	23-item online survey on demographics, physical symptoms, ergonomics, and environment/equipment	17.2 % (54/314)	Female surgeons experience more treatment for their hands (*p* = 0.028). Women with a size 5.5–6.5 surgical glove report discomfort in their shoulder area more commonly than men with the same surgical glove size (*p* = 0.004).

## Data Availability

The data used to support the findings of this study are included within the article and are available on request from the corresponding author.
